# Uncovering the components of the *Francisella tularensis* virulence stealth strategy

**DOI:** 10.3389/fcimb.2014.00032

**Published:** 2014-03-07

**Authors:** Bradley D. Jones, Matthew Faron, Jed A. Rasmussen, Joshua R. Fletcher

**Affiliations:** ^1^Department of Microbiology, The University of Iowa Carver College of MedicineIowa City, IA, USA; ^2^The Genetics Program, The University of Iowa Carver College of MedicineIowa City, IA, USA; ^3^The Midwest Regional Center for Excellence in Biodefense and Emerging Infectious Disease Research, Washington UniversitySt. Louis, MO, USA

**Keywords:** *Francisella tularensis*, FPI virulence genes, phagosome escape, unique LPS, stealth strategy, capsule mutant, virulent Schu S4

## Abstract

Over the last decade, studies on the virulence of the highly pathogenic intracellular bacterial pathogen *Francisella tularensis* have increased dramatically. The organism produces an inert LPS, a capsule, escapes the phagosome to grow in the cytosol (FPI genes mediate phagosomal escape) of a variety of host cell types that include epithelial, endothelial, dendritic, macrophage, and neutrophil. This review focuses on the work that has identified and characterized individual virulence factors of this organism and we hope to highlight how these factors collectively function to produce the pathogenic strategy of this pathogen. In addition, several recent studies have been published characterizing *F. tularensis* mutants that induce host immune responses not observed in wild type *F. tularensis* strains that can induce protection against challenge with virulent *F. tularensis*. As more detailed studies with attenuated strains are performed, it will be possible to see how host models develop acquired immunity to *Francisella*. Collectively, detailed insights into the mechanisms of virulence of this pathogen are emerging that will allow the design of anti-infective strategies.

## Introduction

Bacterial pathogens adopt a variety of different strategies to infect and grow within a chosen host. In general, opportunistic pathogens take advantage of a compromised host environment to employ their virulence factors to generate a productive infection. Overt pathogens, such as *Francisella tularensis*, have developed sophisticated strategies to alter and/or avoid recognition and antimicrobial killing mechanisms to reach or create a privileged growth site within a “normal” or healthy host, causing the disease tularemia. This organism causes disease by diverse routes, including oral, subcutaneous, and pneumonic; the respiratory route is of particular concern because infection with 50 or less organisms is associated with mortality rates of 30–60%, if untreated (Tarnvik and Berglund, 2003; McLendon et al., 2006). Regardless of the route of infection, the virulence strategy of *F. tularensis* involves entering host cells, escaping from the phagosome and growing within the cytosol of the infected host. Surprisingly, the host immune responses to these activities are delayed and significantly less than expected (Jones et al., 2012). Cells infected with *F. tularensis* quickly disseminate throughout the host to the liver and spleen. Uncontrolled growth in systemic organs causes host cell damage, hypersecretion of cytokines and death by “cytokine storm” (Sharma et al., 2011). Due to the extreme virulence and ease of aerosol dissemination of *F. tularensis*, the US Centers for Disease Control and Prevention have classified this organism as a Tier 1 select agent because of its potential development into a bioweapon (Dennis et al., 2001).

Here we review the literature describing the work that is characterizing the intracellular strategy of this organism as well as the interactions that occur between these organisms and a wide variety of different host cell types (Conlan and North, 1992; Forestal et al., 2003; Lindemann et al., 2007; Schulert et al., 2009; Horzempa et al., 2010). We will highlight studies that have used virulent *F. tularensis* strains since it is becoming clear that work with model organisms (i.e., *F. tularensis* subsp. *holarctica* LVS and *F. novicida*) have sometimes provided results that are different than those obtained with the virulent strains, perhaps not surprisingly since LVS is an attenuated vaccine strain. In addition, we discuss recent work in which mutagenesis studies have identified mutants with significantly altered virulence phenotypes that are yielding important insights into the immune evasion mechanisms of virulent *F. tularensis*. We will discuss the virulence roles of the unique *F. tularensis* LPS and capsule and we also report the presence of glycoproteins that carry O-antigen. In addition, we will discuss various aspects of *Francisella* immunology and how attenuated *F. tularensis* strains are providing opportunities to study adaptive immune in a murine model of infection. Finally, we will attempt to provide insights into how this accumulating body of work may direct efforts to develop strains that can provide protective immunity against *F. tularensis* infection.

## Genetic approaches to discover *Fcrancisella tularensis* virulence mechanisms

Efforts to understand the molecular details of the *F. tularensis* virulence strategy have progressed rapidly in the last decade. These research efforts have relied on genetic approaches to identify *Francisella* genes that are required for various aspects of pathogenesis. Qin and Mann (reference) performed transposon mutagenesis in the Schu S4 strain and identified numerous genes required for growth in HepG2 cells, including metabolic genes (i.e., *car* and *pur* genes, *ggt*, etc.) and hypothetical genes that have been found in screens by numerous groups, including our own. Weiss et al. identified *F. novicida* genes required for systemic mouse disease by creating a library of random transposon insertions in the chromosome of *F. novicida* (Weiss et al., 2007). This work identified the genes on the *Francisella* Pathogenicity Island (FPI, required for phagosomal escape), LPS O-antigen biosynthetic genes and 44 previously unidentified genes as important for *F. novicida* virulence. Many of the genes found in these screens are important for modifying the intracellular growth niche of the organism, acquiring nutrients or in modifying the host immune response to the presence of the bacteria.

Our research group has performed a similar screen to identify genes in virulent *F. tularensis* Schu S4 required for growth in human macrophages (Lindemann et al., 2011). Using a transposon system similar to that of Weiss et al., pools of *F. tularensis* Schu S4 mutants were screened for strains that were significantly reduced in their ability to grow within human monocyte-derived macrophages (MDMs). That work identified *F. tularensis* Schu S4 mutants that were defective in production of capsule and O-antigen, defective in FPI gene expression, nutritional mutants, as well as many other mutants in genes with unknown functions. These mutants are yielding important insights into *Francisella* mechanisms of virulence and are providing avenues to study host immune responses to *Francisella* strains that have lost some of their virulence properties.

## Factors important in the uptake of *Francisella tularensis* into phagocytic cells

The virulence of *Francisella* is dependent upon the ability of the organisms to enter, persist and replicate within host cells as strains that have lost these abilities are avirulent (Brotcke and Monack, 2008; Charity et al., 2009). While it is not known which host cells provide growth niches that are essential to *Francisella* virulence, it is known that *Francisella* infects multiple cell types including phagocytes such as MDMs and polymorphonuclear leukocytes (PMN) as well as non-phagocytic epithelial and endothelial cells (Golovliov et al., 2003; Lauriano et al., 2004; Nano et al., 2004; McCaffrey and Allen, 2006; Qin and Mann, 2006; Schulert and Allen, 2006; Lindemann et al., 2007; Moreland et al., 2009). Upon contact with a phagocytic cell, *F. tularensis* can interact with different receptors, depending upon the opsonization state of the organism. In studies performed with non-opsonized *Francisella*, the mannose receptor plays an important role as non-opsonized Schu S4 had reduced uptake into macrophages acquired from mannose receptor knockout mice compared to macrophages from wild type mice (Geier and Celli, 2011). Consistent with this observation, blocking the mannose receptor with antibodies or soluble mannan significantly inhibits *Francisella* uptake (Balagopal et al., 2006; Schulert and Allen, 2006; Geier and Celli, 2011). However, deletion or blockade of the mannose receptor does not completely inhibit *Francisella* uptake, indicating that other receptors are involved in the uptake of the bacteria (Geier and Celli, 2011). While the reasons for the ability of this organism to use some many different receptors for internalization into phagocytic cells is not well understood, it does give the organism the capability to be internalized into a wide variety of different cell types. Entry into cells via different receptors doubtlessly activates different signaling cascades that elicit different cytokine responses from the host cells. Research efforts, by different groups, are aimed at understanding how the use of different receptors for internalization of *F. tularensis* impacts the infection dynamics between the pathogen and the host.

Studies of opsonized *Francisella* have demonstrated that the type of opsonization (serum vs. antibody) can alter the receptor employed for internalization of the organisms. In addition, independent of the method of opsonization, internalization of opsonized *Francisella* is significantly increased compared to non-opsonized organisms (Ben Nasr et al., 2006; Pierini, 2006; Geier and Celli, 2011). The targets of complement opsonization appear to be LPS and capsule, which protect the organism from the killing activity of complement while simultaneously stimulating the uptake of the bacteria into host cells (Clay et al., 2008). Several studies have implicated complement receptors in the uptake of *Francisella* strains (Schulert and Allen, 2006; Clay et al., 2008; Geier and Celli, 2011; Schwartz et al., 2012). Knockout of the CR3 receptor significantly reduces internalization of serum-opsonized Schu S4 in macrophages (Clay et al., 2008; Geier and Celli, 2011) whereas, serum-opsonized LVS utilizes CR4 to enter MDMs and dendritic cells (Ben Nasr et al., 2006; Schwartz et al., 2012). Both *F. tularensis* Schu S4 and LVS (serum opsonized) can use CR1 and CR3 for internalization into neutrophils (Schwartz et al., 2012). Additionally, both strains can utilize scavenger receptor A (SRA) for uptake into MDMs (Pierini, 2006). In contrast, IgG antibody-opsonized organisms have been shown to interact almost exclusively with Fc receptor (FcγR), since MDMs isolated from FcγR knockout mice are significantly reduced in their ability to internalize antibody opsonized Schu S4 (Geier and Celli, 2011). Antibody opsonization of Schu S4 has been associated with production of superoxide and decreased intracellular growth compared to non-opsonized bacteria (Geier and Celli, 2011). These data indicate that *Francisella* has evolved multiple mechanisms of internalization, which appear to be equally important, to take advantage of different host conditions and it is likely that the mechanism of uptake is important role in the subsequent interactions of the organism with its host.

Little is understood of the *Francisella* factors that mediate uptake into host cells. Recent data from our lab has demonstrated that the *Francisella* capsule and/or LPS are important in reducing uptake by phagocytes (Lindemann et al., 2011). Mutants in the *waaY* and *waaL* genes, which produce no capsule and lack the O-antigen side chain, displays ~10 fold increase in uptake into MDMs compared to wild type Schu S4 (Lindemann et al., 2011; Rasmussen et al., 2014). One of these mutants also displays slightly increased uptake into epithelial cells (unpublished observation). The mechanism for this increased uptake is unknown but it is tempting to speculate that mutations that result in disruptions of capsule and LPS biosynthesis either change the charge of the bacterial surface or uncover other surface molecules that mediate uptake. While not much is known about the bacterial factors that facilitate entry of *Francisella* into various host cells, strains that display increased uptake may be valuable in uncovering the mechanism of entry.

## The intracellular interactions of *Francisella tularensis*

After entry, *F. tularensis* is located within a phagosome that begins to mature into a phagolysosome. Although maturation of the phagosome progresses significantly, *Francisella* has the ability to prevent fusion of the phagosome with the lysosome (Anthony et al., 1991; Checroun et al., 2006). During this process, the *Francisella*-containing phagosome (FCP) becomes decorated with both early and late endosomal markers: EEA-1, CD63, LAMP-1, LAMP-2, and Rab5 (Golovliov et al., 2003; Clemens et al., 2004; Checroun et al., 2006; Santic et al., 2006). Instead of fusing with the lysosome, the bacteria degrade the phagosomal membrane and egress into the host cytosol between 1 and 4 h post entry into the cell (Clemens et al., 2004, 2009; Chong et al., 2008). Interestingly, inhibition of phagosomal acidification delays *F. tularensis* escape and replication (Chong et al., 2008). The molecular mechanism that allows the bacteria to escape the phagosome is still uncharacterized but several studies have established that disruption of FPI genes renders the bacteria unable to escape the phagosome (Nano et al., 2004; Barker and Klose, 2007; de Bruin et al., 2007; Nano and Schmerk, 2007; Ludu et al., 2008; Buchan et al., 2009). In *F. tularensis* type A and type B strains both copies of the FPI must be mutated to inhibit phagosomal escape.

As *Francisella* reaches the cytosol the bacteria begin to replicate quickly, with data showing that virulent Schu S4 has an intracellular doubling time of ~1 h (Chong et al., 2008). This intracellular growth appears to last up to 48 h *in vitro*. Depending upon the host cell type, the organisms can replicate 50–1500 fold (Lai et al., 2001; Qin and Mann, 2006; Bonquist et al., 2008; Schulert et al., 2009; Edwards et al., 2010). Eventually host cell resources are consumed and the cell dies, releasing bacteria into the extracellular environment (Lai and Sjostedt, 2003; Lai et al., 2004; Celli and Zahrt, 2013).

Several bacterial factors that are essential for cytosolic growth have been identified through genetic approaches. Many of these genes are involved in metabolic pathways such as purine biosynthesis (*purMCD*) or uracil biosynthesis (*pyrF*) (Pechous et al., 2006, 2008; Horzempa et al., 2010). Recent work using *F. novicida* demonstrated that the *FTN1586* open reading frame, an ortholog of *ansP*, encodes an asparagine transporter that is important for cytosolic growth (Gesbert et al., 2013). Similar roles in cytosolic growth have been identified for other transport proteins in the LVS strain, including, *FTL1645*, *FTL1586*, and *FTL0129* (Marohn et al., 2012). Other genes have been identified that are involved in cytosolic replication including *dipA*, *FTT0989*, *ripA*, and the γ-glutamyl transpeptidase, *ggt* (Brotcke et al., 2006; Fuller et al., 2008; Alkhuder et al., 2009; Wehrly et al., 2009; Chong et al., 2012).

Three genes, *migR*, *trmE*, and *cphA* have been identified from genetic screens and shown to be involved in modulating FPI expression and growth inside of MDMs (Buchan et al., 2009; Charity et al., 2009; Lindemann et al., 2011). It has recently been shown by our research group that each of these genes affects FPI expression by indirectly altering intracellular concentrations of the alarmone ppGpp (Faron et al., 2013). The ppGpp alarmone has been shown to strengthen the interaction of FevR and RNA polymerase to induce expression of FevR-dependent genes (Charity et al., 2009). Despite each of these mutants having reduced ppGpp concentrations, each mutant has a unique growth pattern in different *in vitro* infection models. The LVS Δ*trmE* strain did not grow inside of MDM cells, but replicated in both A549 and HEp-2 cells while the LVS Δ*migR* mutant only replicated in HEp-2 cells. The LVS Δ*cphA* strain was unable to grow in any of the three cell types (Faron et al., 2013). These data indicate that different host cells present different intracellular growth challenges to *Francisella*; the bacteria appear to respond to these challenges via different homeostatic feedback pathways that contribute to the intracellular ppGpp pools.

Evidence is accumulating that significant differences exist between virulent *F. tularensis* strains and LVS. For instance, mutation of *migR* decrease *F. tularensis* LVS virulence for mice but deletion of *migR* in Schu S4 has a very minor effect on mouse virulence (Buchan, 2009). It is possible that this virulence difference is due to steady-state FPI gene expression levels between Schu S4 and LVS, as *iglA*-*lacZ* reporter activity in Schu S4 is about 3-fold higher than that observed in the LVS strain (Faron et al., 2013).

We have recently identified another difference between *F. tularensis* Schu S4 and *F. tularensis* LVS. Using recently immortalized human AT-II cells (ABM), we have compared the ability of virulent Schu S4 and LVS to enter and replicate within these cells. Interestingly, Schu S4 grows ~100-fold better in these airway epithelial cells than does LVS, suggesting that LVS attenuation may be partly due to an inability to effectively grow within epithelial lung cells (manuscript in preparation). To our knowledge, this is the first cell type in which such a stark difference in intracellular growth is observed between Schu S4 and LVS. These results indicate that it may be important to identify host cells that are key growth sites for the bacteria prior to efficient dissemination to distal organs. This knowledge may be vital in efforts to produce live attenuated vaccine strains.

## The atypical lipopolysaccharide of *Francisella tularensis*

An important contributing factor to the high virulence of this pathogen is that its early intracellular growth appears to be unchecked by significant host responses (Bosio, 2011). A factor that is important in *F. tularensis* immune evasion is the lipid A component of LPS. *Francisella* lipid A species are unusual in that they are asymmetrical, tetraacylated and have longer than normal fatty acids chains (16–18 carbons). This is in contrast to the lipid A from most Gram-negative bacteria that contain six acyl chains of 12–14 carbons in length and phosphate groups available for interactions with TLR-4 that stimulate strong proinflammatory responses (Raetz, 1990; Poltorak et al., 1998; Beutler and Poltorak, 2000). For *Francisella*, the non-prime phosphate on the di-glucosamine backbone can be shielded by a galactosamine while the phosphate on the prime side of the sugar backbone is often missing (Vinogradov et al., 2002; Phillips et al., 2004; Gunn and Ernst, 2007). Multiple species of lipid A with some or all of these differences may be present in the outer membrane of a single organism (reference). The differences in the *Francisella* lipid A make the endotoxin of *F. tularensis* unable to bind to LBP and therefore unable to activate TLR-4 signaling pathways, rendering it inert compared to typical endotoxins (Hajjar et al., 2006; Gunn and Ernst, 2007). Several research groups are working to understand how acylation, length of fatty acid side chains and shielding the phosphates of the endotoxin contribute to the lack of bioactivity of the *F. tularensis* LPS (Kanistanon et al., 2012). These studies are providing important insights into how bacteria can modify their LPS to lessen the ability of the host to recognize their presence and activate key host cytokine pathways.

## Characteristics of the *Francisella tularensis* capsule and its role in bacterial virulence

The role of the *Francisella* capsule in immune evasion and pathogenesis is an active area of investigation. Early work aimed at characterization of a crude preparation of the *Francisella* capsule revealed that it was composed of mannose, rhamnose, and dideoxy sugars (Hood, 1977). Sandstrom et al. created an acapsular *Francisella* strain and found that it was more sensitive to antibody-mediated killing (Sandstrom et al., 1988), although unfortunately this strain was not preserved for additional studies. Recent work using a monoclonal antibody directed against purified capsular material of *Francisella* has helped to characterize some aspects of this structure (Apicella et al., 2010). The capsule ranges in size from 100 to 250 kDa and it is present in all type A and B strains of *F. tularensis* that have been examined (Apicella et al., 2010). Immunization of mice with purified capsular material elicited circulating anti-capsule antibodies that protected from challenge with *F. tularensis* LVS but did not protect from *F. tularensis* Schu S4 challenge (Apicella et al., 2010).

Most bacterial capsules are large structures formed of repeating sugar subunits that are held together by glycosidic bonds and these structures associate with the bacterial outer membrane either directly or indirectly. There are four general classes of capsules for Gram-negative bacteria based upon biochemical, genetic, serological, and physical properties (Orskov et al., 1977; Daly et al., 1997). Of particular interest to this discussion of *Francisella* virulence factors are Group 4 capsules. Group 4 capsules are composed of similar (often identical) O-antigen sugars as those found in the LPS, contain acetimido sugars in their repeat unit structures, and are greater than 100 kDa in size (Whitfield, 2006). The capsule of *Francisella* has been shown to have a molecular weight of 100–250 kDa and contain the core sugar tetrasaccharide repeat of <2-acetamido-2,6-dideoxy-o-glucose (o-QuiNAc), 4,6-dideoxy-4-formamido-D-glucose (o-Qui4NFm), and 2-acetamido-2-deoxy-o-galacturnoamide (o-GalNAcAN), with the o-GalNAcAN unit present at twice the concentration of the other two sugars (Apicella et al., 2010; Wang et al., 2011). This is the exact composition of the sugars present in the *Francisella* repeating O-antigen subunits of the LPS (Vinogradov et al., 2002; Thomas et al., 2007). In a report characterizing the purified *Francisella* capsule, it was found that neither lipid A nor 2-keto-3-deoxyoctulsonic acid (KDO) were attached to the purified capsular structure, providing additional support that the LPS and capsule are distinct from each other (Apicella et al., 2010). These observations indicate that the *Francisella* capsule is similar to capsules produced by some strains of the Gram-negative bacteria *Vibrio cholera*, *Escherichia coli*, and *Salmonella enterica* which produce capsules that incorporate the LPS O-antigen (Jayaratne et al., 1993; Zhang et al., 2002; Peleg et al., 2005; Barak et al., 2007; Chen et al., 2007). Work to identify capsule biosynthetic genes has produced mutant strains that are disrupted for both capsule and LPS biosynthesis, indicating that the LPS and capsule biosynthetic pathways share many components for their assembly (unpublished observation). Efforts are still underway to identify the genes responsible for the unique biosynthetic steps in each pathway, such as the anchoring mechanism of capsule to the outer membrane, which is apparently distinct from the lipid A anchor of LPS (Bandara et al., 2011; Lindemann et al., 2011; Rasmussen et al., 2014).

One research group (Zarrella et al., 2011) has published data demonstrating that an alternate non-O-antigen capsule is present when *Francisella* is grown in brain heart infusion media (BHI). This high molecular weight alternate capsule species can be detected by PAGE in a *F. tularensis* LVS *wbtA* mutant that is unable to produce O-antigen. This indicates that this alternate capsule is distinct from the O-antigen capsule our research group has described. However, the composition of this structure was not reported. Another group also described the existence of a high molecular weight capsule-like material present in an *F. tularensis* LVS *wbtI* mutant strain, which is unable to produce O-antigen (Bandara et al., 2011). The authors reported that this capsule-like material was composed of glucose, galactose and mannose sugars. While it is not known if the structures reported by Zarella et al. and Bandara et al. are the same, both appear to be high molecular weight capsule-like molecules and both are present in strains with O-antigen defects.

## *F. tularensis* produces proteins that are glycosylated with the O-antigen

**Figure 1:**
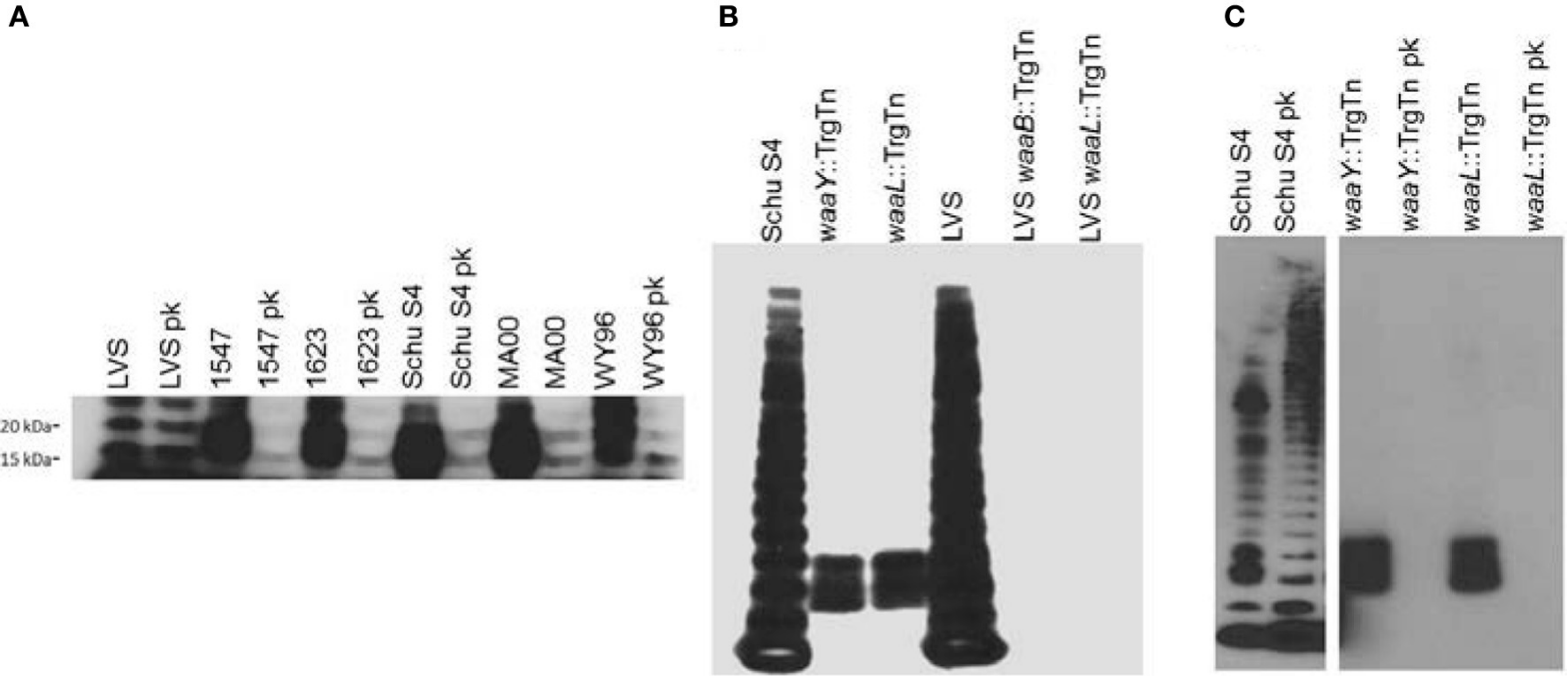
***F. tularensis* strains produce O-antigen glycosylated proteins.** One ml of *F. tularensis* broth cultures was centrifuged at 8000 × g for 2 min before resuspending the pellet in Buffer Part A, (6 mM Tris, 10 mM EDTA, and 2% [wt/vol] sodium dodecyl sulfate [pH 6.8]) and heated to 65°C to sterilize cultures. Bacterial lysates were incubated with, or without, proteinase K (New England Biolabs, Ipswitch, MA) at 37°C for 24 h before lyophilizing. Approximately 14 mg of bacterial material from each sample was mixed with NuPage (Life Technologies, Carlsbad, CA) sample reducing agent and buffer, boiled, and loaded into a 4–12% Bis-Tris NuPage gel and electrophoresed using NuPage MES SDS running buffer (Life Technologies, Carlsbad, CA). For immunoblots, samples were transferred to nitrocellulose and probed with FB11 primary antibody to detect O-antigen attached to LPS or to protein (QED Bioscience, San Diego, CA). Bands were visualized using goat anti-mouse IgG (H+L) conjugated to horseradish peroxidase (Jackson ImmunoResearch, West Grove, PA) and SuperSignal West Pico chemiluminescent substrate (Pierce Biotechnology, Rockford, IL). **(A)** Western blot analysis of LPS preparations from *F. tularensis* LVS, virulent type B *F. tularensis* subsp.*holarctica* strains (1547 and 1623) and virulent type A *F. tularensis* subsp. tularensis strains (Schu S4, MA00, and WY96) using the O-antigen FB11 antibody for detection of bands containing O antigen, without or with proteinase K (pk) treatment. **(B)** Western blot of whole cell lysates of *F. tularensis* Schu S4, *F. tularensis* Schu S4 *waaY*::TrgTn and *F. tularensis Schu S4 waaL*::TrgTn, *F. holarctica* LVS, *F. holarctica* LVS *waaY*::TrgTn and *F. holarctica* LVS *waaL*::TrgTn probed with the anti-O antigen monoclonal antibody FB11 to detect the presence of LPS O antigen laddering in these strains. **(C)** Western blot analysis of *F. tularensis* Schu S4, *F. tularensis* Schu S4 *waaY*::TrgTn and *F. tularensis* Schu S4 *waaL*::TrgTn whole cell lysates without or with (pk) proteinase K treatmenst, using O-antigen FB11 antibody.

Efforts to understand the roles of bacterial glycoproteins have gained momentum from recent data indicating that some bacterial glycoproteins may play roles in host-pathogen interactions (Straskova et al., 2009; Egge-Jacobsen et al., 2011; Balonova et al., 2012). *Francisella* species have recently been shown to produce significant amounts of glycoproteins, many of which appear to be outer membrane proteins (Balonova et al., 2010). We have recently discovered that both virulent type A and B strains of *F. tularensis* produce O-antigen glycoproteins, detectable in whole cell lysates that bind to a monoclonal antibody specific for the O-antigen subunit (FB11, QED Biosciences). Interestingly, the *F. tularensis* LVS strain does not produce detectable quantities of these glycosylated proteins (Figure 1A). Furthermore, when the glycoprotein profiles of Schu S4 LPS O-antigen mutants [*FTT1236* (*waaY*) and *FTT1238c* (*waaB*)] were compared to the orthologous LVS mutants (*FTL0708* and *FTL0706*, respectively), we found that the LVS mutants lacked detectable O-antigen reactive protein bands that are present in the Schu S4 mutants (Figure 1B). Early work with these mutants obscured O-antigen protein reactivity due to proteinase K digestion during sample preparation (Lindemann et al., 2011). More recent work with non-proteinase K treated whole cell lysates has revealed that these strains do produce O-antigen reactive bands which can be detected by Western blots (Figure 1C).

Another group has reported findings that are consistent with these observations. Balonova et al. (2012) reported the presence of a *Francisella* lipoprotein that is glycosylated with O-antigen sugars and that the glycosylated lipoprotein was absent in an *F. tularensis* subsp. *holarctica* FSC200 *wbtDEF* mutant (Balonova et al., 2012). We believe that protein O-antigen glycosylation, unique to virulent *F. tularensis* strains, may play a role in either the virulence of the organism or aid in evading the host immune response. Future work in the lab is focused on understanding the significance of O-antigen glycosylated proteins in *F. tularensis* pathogenesis.

## Differences in adaptive immune responses to virulent *F. tularensis* and *F. tularensis* LVS

One of the key features of the murine pneumonic tularemia model is the rapid time to death (~5 days post infection), which precludes the development of a robust adaptive immune response. This aspect of *Francisella* virulence has made vaccine development a daunting challenge, and many labs have used *F. tularensis* LVS as a less virulent substitute to study aspects of B and T cell biology in sublethal infection and immunization. Evidence is accumulating that the host response to *Francisella* infection is strain-specific and responses that occur to *F. tularensis* LVS or *F. novicida* infection may be different than those observed for virulent *F. tularensis* (i.e., Schu S4) (Kurtz et al., 2013; Laws et al., 2013). This is logical; the LVS strain was purposefully selected as a vaccine strain based upon its attenuated virulence phenotype (Sandstrom, 1994; Elkins et al., 2003). A vaccine against fully virulent *Francisella* strains will almost certainly require stimulation of cellular immunity, though the mechanisms by which these processes can be directed against *Francisella* remain elusive (Ray et al., 2009; Conlan, 2011).

Protective antibodies were an early target of research into immunity to *Francisella*. In studies utilizing a Type A strain, Foshay (1946) showed that transfer of hyperimmune serum from horses or goats provided protection to ~70–90% of rats infected subcutaneously, although the written details of these experiments are vague. More recent studies utilizing the mouse model have found contradictory results (Foshay, 1946; Kirimanjeswara et al., 2008). Kirimanjeswara et al. found that immune serum from LVS-immunized mice did not provide protection against Schu S4. *In vitro*, however, they observed that murine alveolar macrophages stimulated with interferon-γ were able to kill immune serum opsonized Schu S4 (Kirimanjeswara et al., 2008). While the latter result is intriguing, Crane et al. have shown that Schu S4 may be able to avoid this fate *in vivo* by interacting with the host serine protease plasmin. Schu S4 can bind to active plasmin, which can degrade *Francisella*-specific antibodies (Crane et al., 2009). The authors found that while antibody opsonization of both Schu S4 and LVS resulted in an increase in TNF-α, the presence of active plasmin on Schu S4, but not LVS, inhibited the induction of TNF-α in infected cells (Crane et al., 2009). These results highlight a difference between the two strains that may have important implications for how virulent *Francisella* species avoid early detection and control. To that end, plasminogen knockout mice might be an interesting model to examine the role of antibody opsonization and plasmin during Schu S4 infection *in vivo* (Berri et al., 2013).

As with antibodies, the role of B cell activity in providing immunity to *Francisella* species is complex; however, it is clear that mice lacking B cells are less able to control or survive a *Francisella* infection (Crane et al., 2012). Data is emerging that a specific subset of B cells (B1a) can have almost opposite effects in the host response, depending on the infecting strain and the model of infection (Cole et al., 2009; Crane et al., 2013). Cole et al. demonstrated that immunization with lipopolysaccharide from LVS generated a B1a cell-dependent protective response against an intraperitoneal challenge with ~10^3^ CFU of LVS (Cole et al., 2009). In contrast, Crane et al. utilized a short-term low dose antibiotic treatment after intranasal infection with Schu S4 (the convalescent model) and observed that mice largely deficient in B1a cells (XID mice) survived better than did wild type mice (Crane et al., 2013). The increased survival in these mice was found to be associated with a reduction in the anti-inflammatory cytokine IL-10, which is a potent inhibitor of IL-12. The latter cytokine stimulates interferon-γ production, and is necessary for the survival of tularemia (Crane et al., 2012). Curiously, Metzger et al. reported that IL-10^−/−^ mice succumb to standard intranasal Schu S4 infection similar to wild type mice, though it should be noted that the time scales of infection are different between these studies and direct comparisons may not be appropriate (Metzger et al., 2013). The immune responses observed in the convalescent model would not have time to develop in a standard intranasal infection, although it would be of interest to see if IL-10^−/−^ mice phenocopy XID mice in the convalescent model of Schu S4 infection.

One possible target of the anti-inflammatory activity of IL-10 may be at the interface of antigen presenting cells and T lymphocytes. Hunt et al. identified a factor released by *Francisella* infected cells that stimulated IL-10-dependent degradation of MHC class II molecules in macrophages (Hunt et al., 2012). Importantly, supernatants from Schu S4 infected macrophages have also been shown to stimulate the downregulation of MHC class II molecules (Wilson et al., 2009). These data suggest that antigen presentation to CD4^+^ T cells may be reduced *in vivo*. This research group, as well as others, has also shown that *F. novicida* and LVS induce prostaglandin E2 (PGE2) production in infected macrophages (Woolard et al., 2007; Wilson et al., 2009). PGE2 has been shown to inhibit macrophage maturation, and might play a similar role in downregulating pathways important for intracellular killing of *Francisella*, as in *Burkholderia pseudomallei* infections (Zaslona et al., 2012; Asakrah et al., 2013). Interestingly, these groups showed that PGE2 induced by *Francisella* infection inhibited T cell proliferation and interferon-γ production *in vitro*. It has long been known that T cells are important for bacterial control and immunity to *Francisella*; for an excellent summary of this literature, see Cowley and Elkins' 2011 review (Cowley and Elkins, 2011; Crane et al., 2012; Eneslatt et al., 2012). Both CD4^+^ T cells and CD8^+^ T cells were required for survival of a primary Schu S4 infection in the convalescent model of tularemia, but only partial protection against a secondary challenge was observed for wild type mice (66% survival) (Crane et al., 2012). It is intriguing to speculate that the partial protection may be a function of a less than optimal T cell response mediated by PGE2, both in terms of antigen presentation and T cell proliferation. The use of knockout mice lacking various aspects of PGE2 production or signaling may be useful in testing this hypothesis. Our lab has preliminarily identified Schu S4 mutants that provide some protection against wild type Schu S4; it would be interesting to compare PGE2 levels during infection with our strains to those of other groups that have also shown protection (Conlan et al., 2010; Rockx-Brouwer et al., 2012). Additionally, we are developing vectors to express recombinant *Francisella* proteins fused to known CD4^+^ and CD8^+^ epitopes from lymphocytic choriomeningitis virus (LCMV) in an effort to quantify the magnitude of T cell proliferation and maturation state during both the immunization and challenge phases of protection studies.

## Summary

**Figure 2:**
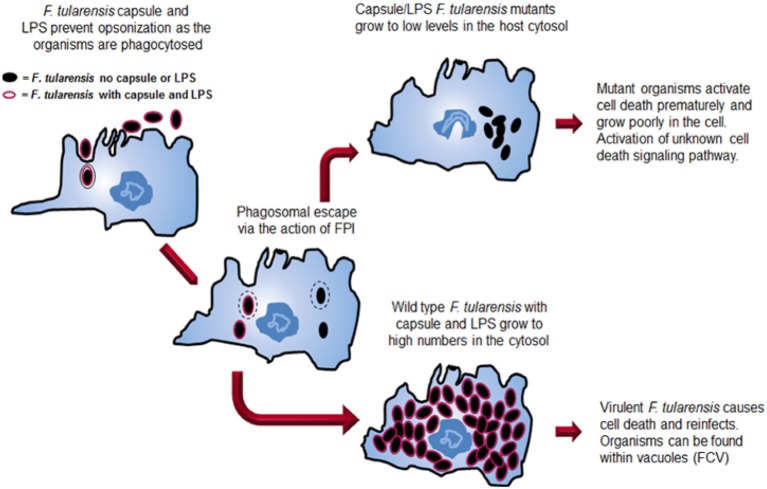
**Comparison of the intracellular growth strategies of wild type *F. tularensis* and a *F. tularensis* capsule/LPS mutant.** Both wild type and mutant organisms are internalized into a host cell, although the absence of capsule and LPS allows increased internalization. Both strains can escape from the phagosome. The wild type strain grows to high numbers in the host cell cytosol before inducing death of the cell. At late stages, organisms can be found in *Francisella*-containing vacuoles (FCVs). In contrast, after mutant organisms escape the phagosome, they grow for a short time in the cytosol before the cell dies preventing additional growth of the organisms.

Significant advances have been made in understanding the virulence of *F. tularensis* over the last decade. The development of genetic tools has been critical in creating *F. tularensis* mutants that are defective in various aspects of virulence. The study of mutants has provided insights into the pathogenic mechanisms of *Francisella* and is pushing forward efforts to understand the mechanisms that this pathogen uses to evade host detection. The virulence strategy of *F. tularensis* has a two-pronged approach (Figure 2). A primary thrust of the *Francisella* virulence strategy is to reach the host cell cytosol as a privileged site for growth. The organism possesses a phagosomal escape mechanism, encoded on the FPI, which allows the organisms to disrupt the phagosomal membrane and enter the cytosol for growth. The FPI genes, which encode the capacity to lyse the phagosomal membrane, are central to the ability of the pathogens to reach the cytosol. Several labs are continuing work aimed at elucidating the molecular details of both the host and bacterial components of this process.

A second component of the *Francisella* virulence strategy employs the capsule and its unique LPS to avoid detection by host innate immunity mechanisms. The capsule and LPS provide protection from the host complement system by protecting the organism from complement opsonization and killing. Perhaps even more importantly, the unique *F. tularensis* LPS provides stealth to the overall virulence strategy as the lipid A does not activate the TLR4 cytokine response pathway.

We have also highlighted in this review some of the differences between laboratory strains of *F. tularensis* and virulent strains of *F. tularensis*. *F. tularensis* Schu S4 and LVS display significant differences in FPI gene expression levels with Schu S4 FPI expression being about 3-fold higher than LVS. Antibodies generated against *F. tularensis* LPS and capsule provide significant protection against murine challenge with *F. tularensis* LVS while little protection against Schu S4 challenge is observed. We also observe that virulent Schu S4 produces significant amounts of proteins glycosylated with O-antigen while LVS has almost undetectable levels of those glycosylated proteins, although the significance of these observations is still unknown. Finally, we have evidence in our lab that LVS does not grow well in a human airway epithelial cell while Schu S4 grows to high levels (manuscript in preparation).

Importantly, there are many research groups actively trying to understand how virulent *F. tularensis* suppresses a variety of host immune responses including neutrophil and macrophage activation mechanisms. The molecular and cellular details of these virulence mechanisms continue to be pursued in detail with the goal of understanding the mechanisms which lead to the extreme virulence of this bacterial pathogen as well as to develop methods to create effective immune responses against *Francisella* infection.

### Conflict of interest statement

The authors declare that the research was conducted in the absence of any commercial or financial relationships that could be construed as a potential conflict of interest.

## References

[B1] Alkhuder K. Meibom K. L. Dubail I. Dupuis M. Charbit A. (2009). Glutathione provides a source of cysteine essential for intracellular multiplication of *Francisella tularensis*. PLoS Pathog. 5:e1000284 10.1371/journal.ppat.1000284 19158962 PMC2629122

[B2] Anthony L. D. Burke R. D. Nano F. E. (1991). Growth of *Francisella spp*. in rodent macrophages. Infect. immun. 59, 3291–3296 1879943 10.1128/iai.59.9.3291-3296.1991 PMC258167

[B3] Apicella M. A. Post D. M. Fowler A. C. Jones B. D. Rasmussen J. A. Hunt J. R. (2010). Identification, characterization and immunogenicity of an O-antigen capsular polysaccharide of *Francisella tularensis*. PLoS ONE 5:e11060 10.1371/journal.pone.0011060 20625403 PMC2897883

[B4] Asakrah S. Nieves W. Mahdi Z. Agard M. Zea A. H. Roy C. J. (2013). Post-exposure therapeutic efficacy of COX-2 inhibition against *Burkholderia pseudomallei*. PLoS Negl. Trop. Dis. 7:e2212 10.1371/journal.pntd.0002212 23675544 PMC3649956

[B5] Balagopal A. MacFarlane A. S. Mohapatra N. Soni S. Gunn J. S. Schlesinger L. S. (2006). Characterization of the receptor-ligand pathways important for entry and survival of *Francisella tularensis* in human macrophages. Infect. Immun. 74, 5114–5125 10.1128/IAI.00795-06 16926403 PMC1594866

[B6] Balonova L. Hernychova L. Mann B. F. Link M. Bilkova Z. Novotny M. V. (2010). Multimethodological approach to identification of glycoproteins from the proteome of *Francisella tularensis*, an intracellular microorganism. J. Proteome Res. 9, 1995–2005 10.1021/pr9011602 20175567 PMC3025813

[B7] Balonova L. Mann B. F. Cerveny L. Alley W. R. Jr. Chovancova E. Forslund A. L. (2012). Characterization of protein glycosylation in *Francisella tularensis* subsp. holarctica: identification of a novel glycosylated lipoprotein required for virulence. Mol. Cell. Proteomics 11:M111015016. 10.1074/mcp.M111.015016 22361235 PMC3394949

[B8] Bandara A. B. Champion A. E. Wang X. Berg G. Apicella M. A. McLendon M. (2011). Isolation and mutagenesis of a capsule-like complex (CLC) from *Francisella tularensis*, and contribution of the CLC to *F. tularensis* virulence in mice. PLoS ONE 6:e19003 10.1371/journal.pone.0019003 21544194 PMC3081320

[B9] Barak J. D. Jahn C. E. Gibson D. L. Charkowski A. O. (2007). The role of cellulose and O-antigen capsule in the colonization of plants by *Salmonella enterica*. Mol. Plant Microbe Interact. 20, 1083–1091 10.1094/MPMI-20-9-1083 17849711

[B10] Barker J. R. Klose K. E. (2007). Molecular and genetic basis of pathogenesis in *Francisella tularensis*. Ann. N.Y. Acad. Sci. 1105, 138–159 10.1196/annals.1409.010 17395737

[B11] Ben Nasr A. Haithcoat J. Masterson J. E. Gunn J. S. Eaves-Pyles T. Klimpel G. R. (2006). Critical role for serum opsonins and complement receptors CR3 (CD11b/CD18) and CR4 (CD11c/CD18) in phagocytosis of *Francisella tularensis* by human dendritic cells (DC): uptake of *Francisella* leads to activation of immature DC and intracellular survival of the bacteria. J. Leukoc. Biol. 80, 774–786 10.1189/jlb.1205755 16857732

[B12] Berri F. Rimmelzwaan G. F. Hanss M. Albina E. Foucault-Grunenwald M. L. Le V. B. (2013). Plasminogen controls inflammation and pathogenesis of influenza virus infections via fibrinolysis. PLoS Pathog. 9:e1003229 10.1371/journal.ppat.1003229 23555246 PMC3605290

[B13] Beutler B. Poltorak A. (2000). Positional cloning of Lps, and the general role of toll-like receptors in the innate immune response. Eur. Cytokine Netw. 11, 143–152 10903793

[B14] Bonquist L. Lindgren H. Golovliov I. Guina T. Sjostedt A. (2008). MglA and Igl proteins contribute to the modulation of *Francisella tularensis* live vaccine strain-containing phagosomes in murine macrophages. Infect. Immun. 76, 3502–3510 10.1128/IAI.00226-08 18474647 PMC2493230

[B15] Bosio C. M. (2011). The subversion of the immune system by *Francisella tularensis*. Front. Microbiol. 2:9 10.3389/fmicb.2011.00009 21687406 PMC3109352

[B16] Brotcke A. Monack D. M. (2008). Identification of *fevR*, a novel regulator of virulence gene expression in *Francisella novicida*. Infect. Immun. 76, 3473–3480 10.1128/IAI.00430-08 18559431 PMC2493208

[B17] Brotcke A. Weiss D. S. Kim C. C. Chain P. Malfatti S. Garcia E. (2006). Identification of MglA-regulated genes reveals novel virulence factors in *Francisella tularensis*. Infect. Immun. 74, 6642–6655 10.1128/IAI.01250-06 17000729 PMC1698089

[B18] Buchan B. W. (2009). Examining the Regulation of Virulence Factors in Francisella tularensis. Ph.D., The University of Iowa

[B19] Buchan B. W. McCaffrey R. L. Lindemann S. R. Allen L. A. Jones B. D. (2009). Identification of migR, a regulatory element of the *Francisella tularensis* live vaccine strain iglABCD virulence operon required for normal replication and trafficking in macrophages. Infect. Immun. 77, 2517–2529 10.1128/IAI.00229-09 19349423 PMC2687360

[B20] Celli J. Zahrt T. C. (2013). Mechanisms of *Francisella tularensis* intracellular pathogenesis. Cold Spring Harb. Perspect. Med. 3:a010314 10.1101/cshperspect.a010314 23545572 PMC3683997

[B21] Charity J. C. Blalock L. T. Costante-Hamm M. M. Kasper D. L. Dove S. L. (2009). Small molecule control of virulence gene expression in *Francisella tularensis*. PLoS Pathog. 5:e1000641 10.1371/journal.ppat.1000641 19876386 PMC2763202

[B22] Checroun C. Wehrly T. D. Fischer E. R. Hayes S. F. Celli J. (2006). Autophagy-mediated reentry of *Francisella tularensis* into the endocytic compartment after cytoplasmic replication. Proc. Natl. Acad. Sci. U.S.A. 103, 14578–14583 10.1073/pnas.0601838103 16983090 PMC1600002

[B23] Chen Y. Bystricky P. Adeyeye J. Panigrahi P. Ali A. Johnson J. A. (2007). The capsule polysaccharide structure and biogenesis for non-O1 *Vibrio cholerae* NRT36S: genes are embedded in the LPS region. BMC Microbiol. 7:20 10.1186/1471-2180-7-20 17362509 PMC1847822

[B24] Chong A. Wehrly T. D. Child R. Hansen B. Hwang S. Virgin H. W. (2012). Cytosolic clearance of replication-deficient mutants reveals *Francisella tularensis* interactions with the autophagic pathway. Autophagy 8, 1342–1356 10.4161/auto.20808 22863802 PMC3442881

[B25] Chong A. Wehrly T. D. Nair V. Fischer E. R. Barker J. R. Klose K. E. (2008). The early phagosomal stage of *Francisella tularensis* determines optimal phagosomal escape and *Francisella* pathogenicity island protein expression. Infect. Immun. 76, 5488–5499 10.1128/IAI.00682-08 18852245 PMC2583578

[B26] Clay C. D. Soni S. Gunn J. S. Schlesinger L. S. (2008). Evasion of complement-mediated lysis and complement C3 deposition are regulated by *Francisella tularensis* lipopolysaccharide O antigen. J. Immunol. 181, 5568–5578 18832715 10.4049/jimmunol.181.8.5568 PMC2782685

[B27] Clemens D. L. Lee B. Y. Horwitz M. A. (2004). Virulent and avirulent strains of *Francisella tularensis* prevent acidification and maturation of their phagosomes and escape into the cytoplasm in human macrophages. Infect. Immun. 72, 3204–3217 10.1128/IAI.72.6.3204-3217.2004 15155622 PMC415696

[B28] Clemens D. L. Lee B. Y. Horwitz M. A. (2009). *Francisella tularensis* phagosomal escape does not require acidification of the phagosome. Infect. Immun. 77, 1757–1773 10.1128/IAI.01485-08 19237528 PMC2681761

[B29] Cole L. E. Yang Y. Elkins K. L. Fernandez E. T. Qureshi N. Shlomchik M. J. (2009). Antigen-specific B-1a antibodies induced by *Francisella tularensis* LPS provide long-term protection against *F. tularensis* LVS challenge. Proc. Natl. Acad. Sci. U.S.A. 106, 4343–4348 10.1073/pnas.0813411106 19251656 PMC2657382

[B30] Conlan J. W. (2011). Tularemia vaccines: recent developments and remaining hurdles. Future Microbiol. 6, 391–405 10.2217/fmb.11.22 21526941

[B31] Conlan J. W. North R. J. (1992). Early pathogenesis of infection in the liver with the facultative intracellular bacteria *Listeria monocytogenes*, *Francisella tularensis*, and *Salmonella typhimurium* involves lysis of infected hepatocytes by leukocytes. Infect. Immun. 60, 5164–5171 1452350 10.1128/iai.60.12.5164-5171.1992 PMC258293

[B32] Conlan J. W. Shen H. Golovliov I. Zingmark C. Oyston P. C. Chen W. (2010). Differential ability of novel attenuated targeted deletion mutants of *Francisella tularensis* subspecies *tularensis* strain SCHU S4 to protect mice against aerosol challenge with virulent bacteria: effects of host background and route of immunization. Vaccine 28, 1824–1831 10.1016/j.vaccine.2009.12.001 20018266 PMC2822029

[B33] Cowley S. C. Elkins K. L. (2011). Immunity to *Francisella*. Front. Microbiol. 2:26 10.3389/fmicb.2011.00026 21687418 PMC3109299

[B34] Crane D. D. Griffin A. J. Wehrly T. D. Bosio C. M. (2013). B1a cells enhance susceptibility to infection with virulent *Francisella tularensis* via modulation of NK/NKT cell responses. J. Immunol. 190, 2756–2766 10.4049/jimmunol.1202697 23378429 PMC3594638

[B35] Crane D. D. Scott D. P. Bosio C. M. (2012). Generation of a convalescent model of virulent *Francisella tularensis* infection for assessment of host requirements for survival of tularemia. PLoS ONE 7:e33349 10.1371/journal.pone.0033349 22428026 PMC3299770

[B36] Crane D. D. Warner S. L. Bosio C. M. (2009). A novel role for plasmin-mediated degradation of opsonizing antibody in the evasion of host immunity by virulent, but not attenuated, *Francisella tularensis*. J. Immunol. 183, 4593–4600 10.4049/jimmunol.0901655 19752236 PMC2748154

[B37] Daly J. M. Jannot C. B. Beerli R. R. Graus-Porta D. Maurer F. G. Hynes N. E. (1997). Neu differentiation factor induces ErbB2 down-regulation and apoptosis of ErbB2-overexpressing breast tumor cells. Cancer Res. 57, 3804–3811 9288791

[B38] de Bruin O. M. Ludu J. S. Nano F. E. (2007). The *Francisella* pathogenicity island protein IglA localizes to the bacterial cytoplasm and is needed for intracellular growth. BMC Microbiol. 7:1 10.1186/1471-2180-7-1 17233889 PMC1794414

[B39] Dennis D. T. Inglesby T. V. Henderson D. A. Bartlett J. G. Ascher M. S. Eitzen E. (2001). Tularemia as a biological weapon: medical and public health management. JAMA 285, 2763–2773 10.1001/jama.285.21.2763 11386933

[B40] Edwards J. A. Rockx-Brouwer D. Nair V. Celli J. (2010). Restricted cytosolic growth of *Francisella tularensis* subsp. *tularensis* by IFN-gamma activation of macrophages. Microbiology 156, 327–339 10.1099/mic.0.031716-0 19926654 PMC2890092

[B41] Egge-Jacobsen W. Salomonsson E. N. Aas F. E. Forslund A. L. Winther-Larsen H. C. Maier J. (2011). O-linked glycosylation of the PilA pilin protein of *Francisella tularensis*: identification of the endogenous protein-targeting oligosaccharyltransferase and characterization of the native oligosaccharide. J. Bacteriol. 193, 5487–5497 10.1128/JB.00383-11 21804002 PMC3187425

[B42] Elkins K. L. Cowley S. C. Bosio C. M. (2003). Innate and adaptive immune responses to an intracellular bacterium, *Francisella tularensis* live vaccine strain. Microbes Infect. 5, 135–142 10.1016/S1286-4579(02)00084-9 12650771

[B43] Eneslatt K. Normark M. Bjork R. Rietz C. Zingmark C. Wolfraim L. A. (2012). Signatures of T cells as correlates of immunity to *Francisella tularensis*. PLoS ONE 7:e32367 10.1371/journal.pone.0032367 22412866 PMC3295757

[B44] Faron M. Fletcher J. R. Rasmussen J. A. Long M. E. Allen L. A. Jones B. D. (2013). The *Francisella tularensis migR*, *trmE*, and *cphA* genes contribute to *F. tularensis* pathogenicity island gene regulation and intracellular growth by modulation of the stress alarmone ppGpp. >Infect. Immun. 81, 2800–2811 10.1128/IAI.00073-13 23716606 PMC3719569

[B45] Forestal C. A. Benach J. L. Carbonara C. Italo J. K. Lisinski T. J. Furie M. B. (2003). *Francisella tularensis* selectively induces proinflammatory changes in endothelial cells. J. Immunol. 171, 2563–2570 12928407 10.4049/jimmunol.171.5.2563

[B46] Foshay L. (1946). A comparative study of the treatment of tularemia with immune serum, hyperimmune serum and streptomycin. Am. J. Med. 1, 180–188 10.1016/0002-9343(46)90036-8 20993803

[B47] Fuller J. R. Craven R. R. Hall J. D. Kijek T. M. Taft-Benz S. Kawula T. H. (2008). RipA, a cytoplasmic membrane protein conserved among *Francisella* species, is required for intracellular survival. Infect. Immun. 76, 4934–4943 10.1128/IAI.00475-08 18765722 PMC2573376

[B48] Geier H. Celli J. (2011). Phagocytic receptors dictate phagosomal escape and intracellular proliferation of *Francisella tularensis*. Infect. Immun. 79, 2204–2214 10.1128/IAI.01382-10 21422184 PMC3125850

[B49] Gesbert G. Ramond E. Rigard M. Frapy E. Dupuis M. Dubail I. (2013). Asparagine assimilation is critical for intracellular replication and dissemination of *Francisella*. Cell. Microbiol. 15, 1–16 10.1111/cmi.12227 24134488

[B50] Golovliov I. Baranov V. Krocova Z. Kovarova H. Sjostedt A. (2003). An attenuated strain of the facultative intracellular bacterium *Francisella tularensis* can escape the phagosome of monocytic cells. Infect. Immun. 71, 5940–5950 10.1128/IAI.71.10.5940-5950.2003 14500514 PMC201066

[B51] Gunn J. S. Ernst R. K. (2007). The structure and function of *Francisella* lipopolysaccharide. Ann. N.Y. Acad. Sci. 1105, 202–218 10.1196/annals.1409.006 17395723 PMC2742961

[B52] Hajjar A. M. Harvey M. D. Shaffer S. A. Goodlett D. R. Sjostedt A. Edebro H. (2006). Lack of *in vitro* and *in vivo* recognition of *Francisella tularensis* subspecies lipopolysaccharide by Toll-like receptors. Infect. Immun. 74, 6730–6738 10.1128/IAI.00934-06 16982824 PMC1698081

[B53] Hood A. M. (1977). Virulence factors of *Francisella tularensis*. J. Hyg. (Lond.) 79, 47–60 10.1017/S0022172400052840 267668 PMC2129918

[B54] Horzempa J. O'Dee D. M. Shanks R. M. Nau G. J. (2010). *Francisella tularensis* Delta*pyrF* mutants show that replication in nonmacrophages is sufficient for pathogenesis *in vivo*. Infect. Immun. 78, 2607–2619 10.1128/IAI.00134-10 20385757 PMC2876533

[B55] Hunt D. Wilson J. E. Weih K. A. Ishido S. Harton J. A. Roche P. A. (2012). *Francisella tularensis* elicits IL-10 via a PGE(2)-inducible factor, to drive macrophage MARCH1 expression and class II down-regulation. PLoS ONE 7:e37330 10.1371/journal.pone.0037330 22615981 PMC3355121

[B56] Jayaratne P. Keenleyside W. J. MacLachlan P. R. Dodgson C. Whitfield C. (1993). Characterization of *rcsB* and *rcsC* from *Escherichia coli* O9:K30:H12 and examination of the role of the rcs regulatory system in expression of group I capsular polysaccharides. J. Bacteriol. 175, 5384–5394 8366025 10.1128/jb.175.17.5384-5394.1993 PMC206593

[B57] Jones C. L. Napier B. A. Sampson T. R. Llewellyn A. C. Schroeder M. R. Weiss D. S. (2012). Subversion of host recognition and defense systems by *Francisella* *spp*. Microbiol. Mol. Biol. Rev. 76, 383–404 10.1128/MMBR.05027-11 22688817 PMC3372254

[B58] Kanistanon D. Powell D. A. Hajjar A. M. Pelletier M. R. Cohen I. E. Way S. S. (2012). Role of *Francisella* lipid A phosphate modification in virulence and long-term protective immune responses. Infect. Immun. 80, 943–951 10.1128/IAI.06109-11 22215738 PMC3294632

[B59] Kirimanjeswara G. S. Olmos S. Bakshi C. S. Metzger D. W. (2008). Humoral and cell-mediated immunity to the intracellular pathogen *Francisella tularensis*. Immunol. Rev. 225, 244–255 10.1111/j.1600-065X.2008.00689.x 18837786 PMC4871322

[B60] Kurtz S. L. Foreman O. Bosio C. M. Anver M. R. Elkins K. L. (2013). Interleukin-6 is essential for primary resistance to *Francisella tularensis* live vaccine strain infection. Infect. Immun. 81, 585–597 10.1128/IAI.01249-12 23230288 PMC3553820

[B61] Lai X. H. Golovliov I. Sjostedt A. (2001). *Francisella tularensis* induces cytopathogenicity and apoptosis in murine macrophages via a mechanism that requires intracellular bacterial multiplication. Infect. Immun. 69, 4691–4694 10.1128/IAI.69.7.4691-4694.2001 11402018 PMC98551

[B62] Lai X. H. Golovliov I. Sjostedt A. (2004). Expression of IglC is necessary for intracellular growth and induction of apoptosis in murine macrophages by *Francisella tularensis*. Microb. Pathog. 37, 225–230 10.1016/j.micpath.2004.07.002 15519043

[B63] Lai X. H. Sjostedt A. (2003). Delineation of the molecular mechanisms of *Francisella tularensis*-induced apoptosis in murine macrophages. Infect. Immun. 71, 4642–4646 10.1128/IAI.71.8.4642-4646.2003 12874344 PMC165996

[B64] Lauriano C. M. Barker J. R. Yoon S. S. Nano F. E. Arulanandam B. P. Hassett D. J. (2004). MglA regulates transcription of virulence factors necessary for *Francisella tularensis* intraamoebae and intramacrophage survival. Proc. Natl. Acad. Sci. U.S.A. 101, 4246–4249 10.1073/pnas.0307690101 15010524 PMC384726

[B65] Laws T. R. Clark G. D'Elia R. V. (2013). Differential role for interleukin-6 during *Francisella tularensis* infection with virulent and vaccine strains. Infect. Immun. 81, 3055–3056 10.1128/IAI.00234-13 23716611 PMC3719598

[B66] Lindemann S. R. McLendon M. K. Apicella M. A. Jones B. D. (2007). An *in vitro* model system used to study adherence and invasion of *Francisella tularensis* live vaccine strain in nonphagocytic cells. Infect. Immun. 75, 3178–3182 10.1128/IAI.01811-06 17339345 PMC1932879

[B67] Lindemann S. R. Peng K. Long M. E. Hunt J. R. Apicella M. A. Monack D. M. (2011). *Francisella tularensis* Schu S4 O-antigen and capsule biosynthesis gene mutants induce early cell death in human macrophages. Infect. Immun. 79, 581–594 10.1128/IAI.00863-10 21078861 PMC3028865

[B68] Ludu J. S. de Bruin O. M. Duplantis B. N. Schmerk C. L. Chou A. Y. Elkins K. L. (2008). The *Francisella* pathogenicity island protein PdpD is required for full virulence and associates with homologues of the type VI secretion system. J. Bacteriol. 190, 4584–4595 10.1128/JB.00198-08 18469101 PMC2446798

[B69] Marohn M. E. Santiago A. E. Shirey K. A. Lipsky M. Vogel S. N. Barry E. M. (2012). Members of the *Francisella tularensis* phagosomal transporter subfamily of major facilitator superfamily transporters are critical for pathogenesis. Infect. Immun. 80, 2390–2401 10.1128/IAI.00144-12 22508856 PMC3416476

[B70] McCaffrey R. L. Allen L. A. (2006). *Francisella tularensis* LVS evades killing by human neutrophils via inhibition of the respiratory burst and phagosome escape. J. Leukoc. Biol. 80, 1224–1230 10.1189/jlb.0406287 16908516 PMC1828114

[B71] McLendon M. K. Apicella M. A. Allen L. A. (2006). *Francisella tularensis*: taxonomy, genetics, and immunopathogenesis of a potential agent of biowarfare. Annu. Rev. Microbiol. 60, 167–185 10.1146/annurev.micro.60.080805.142126 16704343 PMC1945232

[B72] Metzger D. W. Salmon S. L. Kirimanjeswara G. (2013). Differing effects of interleukin-10 on cutaneous and pulmonary *Francisella tularensis* live vaccine strain infection. Infect. Immun. 81, 2022–2027 10.1128/IAI.00024-13 23529615 PMC3676042

[B73] Moreland J. G. Hook J. S. Bailey G. Ulland T. Nauseef W. M. (2009). *Francisella tularensis* directly interacts with the endothelium and recruits neutrophils with a blunted inflammatory phenotype. Am. J. Physiol. Lung Cell. Mol. Physiol. 296, L1076–L1084 10.1152/ajplung.90332.2008 19346432 PMC2692798

[B74] Nano F. E. Schmerk C. (2007). The *Francisella* pathogenicity island. Ann. N.Y. Acad. Sci. 1105, 122–137 10.1196/annals.1409.000 17395722

[B75] Nano F. E. Zhang N. Cowley S. C. Klose K. E. Cheung K. K. Roberts M. J. (2004). A *Francisella tularensis* pathogenicity island required for intramacrophage growth. J. Bacteriol. 186, 6430–6436 10.1128/JB.186.19.6430-6436.2004 15375123 PMC516616

[B76] Orskov I. Orskov F. Jann B. Jann K. (1977). Serology, chemistry, and genetics of O and K antigens of *Escherichia coli*. Bacteriol. Rev. 41, 667–710 334154 10.1128/br.41.3.667-710.1977 PMC414020

[B77] Pechous R. Celli J. Penoske R. Hayes S. F. Frank D. W. Zahrt T. C. (2006). Construction and characterization of an attenuated purine auxotroph in a *Francisella tularensis* live vaccine strain. Infect. Immun. 74, 4452–4461 10.1128/IAI.00666-06 16861631 PMC1539594

[B78] Pechous R. D. McCarthy T. R. Mohapatra N. P. Soni S. Penoske R. M. Salzman N. H. (2008). A *Francisella tularensis* Schu S4 purine auxotroph is highly attenuated in mice but offers limited protection against homologous intranasal challenge. PLoS ONE 3:e2487 10.1371/journal.pone.0002487 18575611 PMC2429968

[B79] Peleg A. Shifrin Y. Ilan O. Nadler-Yona C. Nov S. Koby S. (2005). Identification of an *Escherichia coli* operon required for formation of the O-antigen capsule. J. Bacteriol. 187, 5259–5266 10.1128/JB.187.15.5259-5266.2005 16030220 PMC1196049

[B80] Phillips N. J. Schilling B. McLendon M. K. Apicella M. A. Gibson B. W. (2004). Novel modification of lipid A of *Francisella tularensis*. Infect. Immun. 72, 5340–5348 10.1128/IAI.72.9.5340-5348.2004 15322031 PMC517411

[B81] Pierini L. M. (2006). Uptake of serum-opsonized *Francisella tularensis* by macrophages can be mediated by class A scavenger receptors. Cell. Microbiol. 8, 1361–1370 10.1111/j.1462-5822.2006.00719.x 16882038

[B82] Poltorak A. Smirnova I. He X. Liu M. Y. Van Huffel C. McNally O. (1998). Genetic and physical mapping of the Lps locus: identification of the toll-4 receptor as a candidate gene in the critical region. Blood Cells Mol. Dis. 24, 340–355 10.1006/bcmd.1998.0201 10087992

[B83] Qin A. Mann B. J. (2006). Identification of transposon insertion mutants of *Francisella tularensis tularensis* strain Schu S4 deficient in intracellular replication in the hepatic cell line HepG2. BMC Microbiol. 6:69 10.1186/1471-2180-6-69 16879747 PMC1557513

[B84] Raetz C. R. (1990). Biochemistry of endotoxins. Annu. Rev. Biochem. 59, 129–170 10.1146/annurev.bi.59.070190.001021 1695830

[B85] Rasmussen J. A. Lindemann S. R. Post D. M. B. Gibson B. W. Apicella M. A. Meyerholz D. K. (2014). *Francisella tularensis* Schu S4 capsule and O-antigen mutants are attenuated in a mouse model of Tularemia. Infect. Immun. 82 [Epub ahead of print]. 10.1128/IAI.01640-13 24452684 PMC3993386

[B86] Ray H. J. Cong Y. Murthy A. K. Selby D. M. Klose K. E. Barker J. R. (2009). Oral live vaccine strain-induced protective immunity against pulmonary *Francisella tularensis* challenge is mediated by CD4+ T cells and antibodies, including immunoglobulin A. Clin. Vaccine Immunol. 16, 444–452 10.1128/CVI.00405-08 19211773 PMC2668291

[B87] Rockx-Brouwer D. Chong A. Wehrly T. D. Child R. Crane D. D. Celli J. (2012). Low dose vaccination with attenuated *Francisella tularensis* strain SchuS4 mutants protects against tularemia independent of the route of vaccination. PLoS ONE 7:e37752 10.1371/journal.pone.0037752 22662210 PMC3360632

[B88] Sandstrom G. (1994). The tularaemia vaccine. J. Chem. Technol. Biotechnol. 59, 315–320 10.1002/jctb.280590402 7764815

[B89] Sandstrom G. Lofgren S. Tarnvik A. (1988). A capsule-deficient mutant of *Francisella tularensis* LVS exhibits enhanced sensitivity to killing by serum but diminished sensitivity to killing by polymorphonuclear leukocytes. Infect. Immun. 56, 1194–1202 3356465 10.1128/iai.56.5.1194-1202.1988 PMC259783

[B90] Santic M. Molmeret M. Klose K. E. Abu Kwaik Y. (2006). *Francisella tularensis* travels a novel, twisted road within macrophages. Trends Microbiol. 14, 37–44 10.1016/j.tim.2005.11.008 16356719

[B91] Schulert G. S. Allen L. A. (2006). Differential infection of mononuclear phagocytes by *Francisella tularensis*: role of the macrophage mannose receptor. J. Leukoc. Biol. 80, 563–571 10.1189/jlb.0306219 16816147 PMC1865506

[B92] Schulert G. S. McCaffrey R. L. Buchan B. W. Lindemann S. R. Hollenback C. Jones B. D. (2009). *Francisella tularensis* genes required for inhibition of the neutrophil respiratory burst and intramacrophage growth identified by random transposon mutagenesis of strain LVS. Infect. Immun. 77, 1324–1336 10.1128/IAI.01318-08 19204089 PMC2663180

[B93] Schwartz J. T. Barker J. H. Long M. E. Kaufman J. McCracken J. Allen L. A. (2012). Natural IgM mediates complement-dependent uptake of *Francisella tularensis* by human neutrophils via complement receptors 1 and 3 in nonimmune serum. J. Immunol. 189, 3064–3077 10.4049/jimmunol.1200816 22888138 PMC3436988

[B94] Sharma J. Mares C. A. Li Q. Morris E. G. Teale J. M. (2011). Features of sepsis caused by pulmonary infection with *Francisella tularensis* Type A strain. Microb. Pathog. 51, 39–47 10.1016/j.micpath.2011.03.007 21440052 PMC3090489

[B95] Straskova A. Pavkova I. Link M. Forslund A. L. Kuoppa K. Noppa L. (2009). Proteome analysis of an attenuated *Francisella tularensis* dsbA mutant: identification of potential DsbA substrate proteins. J. Proteome Res. 8, 5336–5346 10.1021/pr900570b 19799467

[B96] Tarnvik A. Berglund L. (2003). Tularaemia. Eur. Respir. J. 21, 361–373 10.1183/09031936.03.00088903 12608453

[B97] Thomas R. M. Titball R. W. Oyston P. C. Griffin K. Waters E. Hitchen P. G. (2007). The immunologically distinct O antigens from *Francisella tularensis* subspecies tularensis and *Francisella novicida* are both virulence determinants and protective antigens. Infect. Immun. 75, 371–378 10.1128/IAI.01241-06 17074846 PMC1828428

[B98] Vinogradov E. Perry M. B. Conlan J. W. (2002). Structural analysis of *Francisella tularensis* lipopolysaccharide. Eur. J. Biochem. 269, 6112–6118 10.1046/j.1432-1033.2002.03321.x 12473106

[B99] Wang Q. Shi X. Leymarie N. Madico G. Sharon J. Costello C. E. (2011). A typical preparation of *Francisella tularensis* O-antigen yields a mixture of three types of saccharides. Biochemistry 50, 10941–10950 10.1021/bi201450v 22091710 PMC3238095

[B100] Wehrly T. D. Chong A. Virtaneva K. Sturdevant D. E. Child R. Edwards J. A. (2009). Intracellular biology and virulence determinants of *Francisella tularensis* revealed by transcriptional profiling inside macrophages. Cell. Microbiol. 11, 1128–1150 10.1111/j.1462-5822.2009.01316.x 19388904 PMC2746821

[B101] Weiss D. S. Brotcke A. Henry T. Margolis J. J. Chan K. Monack D. M. (2007). *In vivo* negative selection screen identifies genes required for *Francisella* virulence. Proc. Natl. Acad. Sci. U.S.A. 104, 6037–6042 10.1073/pnas.0609675104 17389372 PMC1832217

[B102] Whitfield C. (2006). Biosynthesis and assembly of capsular polysaccharides in *Escherichia coli*. Annu. Rev. Biochem. 75, 39–68 10.1146/annurev.biochem.75.103004.142545 16756484

[B103] Wilson J. E. Katkere B. Drake J. R. (2009). *Francisella tularensis* induces ubiquitin-dependent major histocompatibility complex class II degradation in activated macrophages. Infect. Immun. 77, 4953–4965 10.1128/IAI.00844-09 19703975 PMC2772548

[B104] Woolard M. D. Wilson J. E. Hensley L. L. Jania L. A. Kawula T. H. Drake J. R. (2007). *Francisella tularensis*-infected macrophages release prostaglandin E2 that blocks T cell proliferation and promotes a Th2-like response. J. Immunol. 178, 2065–2074 17277110 10.4049/jimmunol.178.4.2065

[B105] Zarrella T. M. Singh A. Bitsaktsis C. Rahman T. Sahay B. Feustel P. J. (2011). Host-adaptation of *Francisella tularensis* alters the bacterium's surface-carbohydrates to hinder effectors of innate and adaptive immunity. PLoS ONE 6:e22335 10.1371/journal.pone.0022335 21799828 PMC3142145

[B106] Zaslona Z. Serezani C. H. Okunishi K. Aronoff D. M. Peters-Golden M. (2012). Prostaglandin E2 restrains macrophage maturation via E prostanoid receptor 2/protein kinase A signaling. Blood 119, 2358–2367 10.1182/blood-2011-08-374207 22234697 PMC3311259

[B107] Zhang Y. L. Arakawa E. Leung K. Y. (2002). Novel *Aeromonas hydrophila* PPD134/91 genes involved in O-antigen and capsule biosynthesis. Infect. Immun. 70, 2326–2335 10.1128/IAI.70.5.2326-2335.2002 11953367 PMC127894

